# Time to rethink the law on part-human chimeras

**DOI:** 10.1093/jlb/lsz005

**Published:** 2019-05-15

**Authors:** Julian J Koplin, Julian Savulescu

**Affiliations:** 1 Biomedical Ethics Research Group, Murdoch Children's Research Institute, Parkville VIC 3052, Australia; 2 Melbourne Law School, Carlton VIC 3053, Australia; 3 Oxford Uehiro Centre for Practical Ethics, Oxford OX1 1PT, United Kingdom

**Keywords:** stem cell research and practice, chimera, moral status

## Abstract

It may soon be possible to generate human tissues and organs inside of part-human chimeras via a technique known as interspecies blastocyst complementation. Using Australian legislation as a case study, we show why this technique of creating part-human chimeras falls within the gaps of existing legislation. We give an overview of the key ethical issues raised by part-human chimera research, and we describe how well these issues are met by a range of possible regulatory approaches. We ultimately argue that regulation of part-human chimera research should be (re)designed to balance two key aims: to facilitate ethical research involving part-human chimeras and to prevent unethical experimentation with chimeras that have an uncertain—and potentially substantial—degree of moral status.

It may soon be possible to generate human tissues and organs inside of part-human chimeras via a technique known as interspecies blastocyst complementation. This technique involves editing the genes of an animal embryo to prevent it from growing specific organs or tissues, creating a ‘developmental niche’ within which human pluripotent cells can generate functional human organs or tissues. Human pluripotent stem cells would then be introduced to the embryo to create a part-human chimera—ie a creature comprised of a mix of human and animal cells.^1^ Most of the chimeric animal's tissues would be comprised of a mix of (primarily) animal and (some) human cells. However, the targeted organ(s) would be mostly or entirely made up of human cells.^2^

Interspecies blastocyst complementation has already been used to generate organs from one species within the body of another. In a landmark 2010 study, Kobayashi and colleagues injected rat pluripotent stem cells into the blastocysts of mice that would otherwise lack the ability to generate a pancreas, without which they would die shortly after birth. However, the rat stem cells successfully filled this developmental niche, generating a functional pancreas almost entirely composed of rat cells within the body of a rat-mouse chimera.^3^ More recently, interspecies blastocyst complementation has been used to generate mouse pancreata inside of mouse-rat chimeras; these pancreata were then successfully transplanted into non-chimeric mice.^4^ In 2017, researchers from the Salk Institute for Biological Studies announced that they had created chimeric human-pig fetuses. These chimeric fetuses were created by injecting human induced pluripotent stem (iPS) cells into pig embryos, which were them implanted into a sow and allowed to develop for 28 days. By the end of this process, human cells could be found throughout multiple tissues of the human-pig chimeric fetuses (albeit at a low rate), suggesting that interspecies blastocyst complementation could potentially be used to generate human organs inside of part-human chimeric animals.^5^

These breakthroughs have been met with excitement, and for good reason. Generating human organs inside of human-pig chimeras would not only provide a novel means of reducing the current shortage of transplantable organs, but could also circumvent the need for (and burdens of) lifelong immunosuppression; iPS cells could be derived from the patient themselves to create organs that are genetically matched with their intended recipient, and would therefore not provoke an immune response.^6^ However, these breakthroughs have also reignited long-running ethical debates. Like other forms of xenotransplantation, interspecies blastocyst complementation raises issues of animal welfare, animal rights, resource allocation in healthcare, and the potential risk of disease transmission from animals to humans.^7^ Unlike other forms of xenotransplantation, generating human organs inside of part-human chimeras also raises difficult questions about the moral status of creatures that are neither fully animal nor fully human.^8^

Interestingly, in many jurisdictions—including some that take an extremely restrictive approach to the creation of chimeric embryos—the creation of part-human chimeras *by introducing human cells to animal embryos* falls between the gaps of existing legislation. Using Australian legislation as a case study, we show how existing legislative frameworks do not specifically regulate the creation of part-human chimeras by introducing human cells to animal embryos. We then evaluate the key ethical concerns associated with creating such chimeras and consider how existing laws on chimera research might be revised. Next, we consider how forms of oversight beyond legislation could protect against unethical forms of research. We ultimately argue that regulation of part-human chimeras should strike a balance between two important goals: to facilitate beneficial research and to prevent unethical experimentation with chimeric animals that possess a substantial degree of moral status.

## CASE STUDY: AUSTRALIAN LEGISLATION ON CHIMERIC EMBRYOS

In Australia, the creation of part-human chimeras is regulated under the Prohibition of Human Cloning for Reproduction (PHCR) Act 2002.^9^ The PHCR Act prohibits both the creation of chimeric embryos and the development of a hybrid embryo for more than 14 days. This would seem to rule out any experimentation with interspecies blastocyst complementation, let alone the generation of human organs inside of live-born part-human chimeras. Interestingly, however, part-human chimeras created by introducing human stem cells to animal embryos fall outside the scope of the PHCR Act's definitions of both chimeric and hybrid embryos.

The PHCR Act defines *chimeric embryos* as ‘a human embryo into which a cell, or any component part of a cell, of an animal has been introduced.’^10^ The human-pig chimeras recently created by the Salk team would not meet the PHCR Act's definition of a chimeric embryo, as in this case human stem cells were added to animal embryos rather than the other way around. The PHCR Act defines *hybrid embryos* as embryos that have been created by the fertilization of a human egg by animal sperm, the fertilization of an animal egg by human sperm, or via nuclear transfer between animal and human eggs.^11^ Hybrid embryos are distinct from chimeric embryos, which cannot be created in any of these ways. Unlike part-human chimeras, each cell of a hybrid embryo would contain a mix of human and animal DNA. By contrast, part-human chimeric embryos comprise a mix of fully animal cells and fully human cells.

Although the PHCR Act prohibits the creation of chimeric embryos, the creation of part-animal chimeric embryos *via introducing human stem cells to animal embryos* falls within the gaps of the existing legislation. Although it is a criminal offense to create an animal-to-human chimera or allow a hybrid embryo to develop beyond 14 days, one could theoretically create a chimeric embryo using human stem cells and bring the resulting animal to term without apparently running afoul of the PHCR Act. The situation in Australia is not unique; other jurisdictions also have laws that fail to address the possibility of creating chimeras by introducing human stem cells into nonhuman animals (Table 1), perhaps because such laws were often designed to address issues around the use of human embryos in research rather than the use of human stem cells more generally. Given recent scientific developments, there is a pressing need to carefully consider the ethics and regulation of part-human chimera research.

**Table 1.** International legislation and commentary on part-human chimeric embryos.

Jurisdiction	Legislation, guidelines, and commentary
Australia	The Prohibition of Human Cloning for Reproduction Act prohibits the creation of chimeric embryos via the introduction of animal cells into human embryos, but not via the introduction of human cells into animal embryos.^16^
Canada	The Assisted Human Reproduction Act 2004 prohibits the creation of chimeras, which are defined as (human) embryos into which cells from other animals or humans have been introduced; it does not prohibit the creation of chimeric embryos by introducing human cells to animal embryos.^17^ However, the main agencies responsible for funding scientific research in Canada expressly prohibit the creation of either form of chimeric embryo, effectively blocking any such research.^18^
USA	Although federal laws do not restrict the creation of part-human chimeras, the National Institutes of Health has issued a moratorium on federal funding for human-animal chimera research as it considers ethical issues associated with the introduction of human stem cells to animal embryos.^19^ The National Academy of Sciences has recommended subjecting part-human chimera research to specialized review in cases where there is a significant possibility that human pluripotent stem cells will contribute to neural or gametic cells and tissues,^20^ while bills introduced (but not passed) in Senate in 2005^21^ and in House in 2016^22^ would have prohibited the creation of several kinds of part-human chimeras, including chimeras whose neural tissues are predominantly human.
UK	The Human Fertilisation and Embryology Act 2008 prohibits (inter alia) keeping a human admixed embryo for longer than 14 days or beyond the appearance of the primitive streak, as well as placing a ‘human admixed embryo’ in an animal to develop. ‘Human admixed embryos’ include human embryos altered by the introduction of one or more animal cells, as well as embryos containing both human and animal DNA in which the animal DNA is not predominant.^23^ The Academy of Medical Sciences has recommended subjecting some categories of chimera research to additional review by an expert body (including research that would make brain function, behavior, or physical appearance more ‘human-like’) while rejecting some categories of research outright (including research involving the creation of
	human–nonhuman primate chimeras with ‘human-like’ brain function or the breeding of animals with human-derived germ cells).^24^
Japan	Under Japanese law human-animal chimeric embryos may only be cultured until the appearance of the primitive streak; they may not be transferred into a human or animal uterus.^25^ The Japanese Expert Panel on Bioethics has recommended repealing these prohibitions and proscribing a narrower range of practices, such as the generation of human brains in human–nonhuman primate chimeras.^26^
Germany	The Embryo Protection Act 1990 prohibits the creation of chimeras via introducing animal cells to a human embryo or fusing human and animal embryos.^27^ The German Ethics Council has recommended prohibiting some additional kinds of chimera research (including the creation of chimeras capable of forming human gametes) and placing additional restrictions on the creation of human-animal brain chimeras, particularly where such research involves nonhuman primates.^28^
France	French law prohibits the creation of chimeric human embryos. However, it is arguably unclear whether the law bans the introduction of human cells to animal embryos, or whether it bans only the introduction of animal cells to human embryos.^29^
Switzerland	Swiss law on assisted reproduction forbids the creation of most kinds of chimeras, including chimeras created by introducing human embryonic stem cells to an animal embryo. However, because the Swiss law did not anticipate the possibility of creating chimeras by introducing human iPS cells to animal embryos, this technique falls within a loophole of the existing legislation.^30^
International guidelines	The International Society for Stem Cell Research (ISSCR) Guidelines for Stem Cell Research and Clinical Translation recommend that part-human chimera research should not be pursued if it involves breeding part-human chimeras with the potential to form human gametes.^31^ The guidelines also hold that research involving chimerism of either the central nervous system or germ line should be subject to specialized research oversight to address possible animal welfare issues.^32^

## ETHICAL IMPLICATIONS OF CREATING PART-HUMAN CHIMERAS

A recent commentary in *Nature Biotechnology* has laid out some of the key ethical issues associated with part-human chimera research, as well as other areas of experimentation involving human- or human-like brains. These include questions about which (if any) kinds of chimera research would unethically blur the lines between humans and animals, whether researchers or others should be able to ‘own’ part-human chimeras, how the welfare of chimeras with humanized brains should be protected, and whether chimeric animals that have developed advanced cognitive capacities should be given special treatment rather than destroyed at the end of a study.^12^ The authors ultimately recommend developing an ethical framework while this area of research remains in an early stage of development.^13^

What ethical objections might be raised against part-human chimera research? After the Salk team announced their creation of human-pig chimeric fetuses, newspaper coverage of the breakthrough highlighted concerns about the ‘unnaturalness’ of creating part-human chimeras, reservations about ‘playing God’, concerns about blurring species boundaries, concerns about animal welfare, and concerns about the uncertain moral status of live-born part-human chimeras, especially in cases where human cells contribute to chimeric animals’ brain.^14^ This range of topics largely mirrors the key points of discussion in the broader bioethics literature on part-human chimeras.^15^

Some of the arguments against creating human–nonhuman chimeras are unpersuasive. Consider the concern that blurring species boundaries is ‘unnatural’ and/or constitutes ‘playing God’ in some pejorative sense. Medical interventions such as vaccines and antibiotics are also unnatural, and we ‘play God’ whenever we develop, prescribe, or use them. An injunction against interfering with nature not only rules out part-human chimera research, but also requires, implausibly, that we reject much of modern medicine. A more moderate version of the unnaturalness or ‘playing God’ objection warns that novel technologies can be dangerous, as their novelty may make it difficult to predict the possible negative consequences of using them.^33^ But this version of the objection does not require us to reject ‘unnatural’ technologies outright; it recommends only that we take care to anticipate and minimize their possible risks.

It is sometimes argued that because part-human chimeras blur the boundaries between human and nonhuman animals, their creation will undermine an important moral view: that it matters, morally, which species we belong to. This argument assumes that we ought to preserve the view that boundaries between species are fixed, natural, and morally relevant, with full moral status reserved exclusively for humans.^34^ Yet even if creating part-human chimeras alters public attitudes in this way (and it is not obvious that it would), it is unclear *why* it is important for us to preserve the view that biological humanness is both necessary and sufficient for full moral status. Indeed, there are good philosophical reasons to think that moral status is conferred not by species membership *per se*, but rather by some set of morally relevant capacities, such as sentience, autonomy, and/or self-consciousness.^35^ This philosophical point has important practical implications. It is possible that misguided beliefs in the moral significance of species membership has led us to underestimate the moral status of (at least some kinds of) nonhuman animals. Underestimating animals’ moral status could, in turn, lead us to accept practices that are deeply unethical.^36^ Accordingly, it might be a good thing if the creation of part-human chimeras prompts us to rethink the significance we currently attach to species membership.

It is possible that scientists could, intentionally or by accident, create chimeras capable of producing human gametes. Accordingly, there might be some risk that sex between chimeric animals could result in human pregnancy. Interestingly, it has been argued that human pregnancy is not *inherently* unethical; it is difficult to pinpoint the source of wrongfulness if (a) the pregnancy does not result in morally unacceptable harm to the chimeric animal, and (b) the human conceptus does not develop beyond the threshold at which it acquires moral status or—in the unlikely scenario a human pregnancy can be brought to term—the human person can go on to live a sufficiently good life.^37^ In any case, there are relatively straightforward measures that could be taken to protect against human pregnancy, such as routinely sterilizing chimeric animals, creating chimeras of only one sex, or physically segregating male and female chimeras.^38^

Research involving live-born chimeric animals raises at least two kinds of animal welfare concerns. First, chimeric animals might be prone to biological dysfunction, which could contribute to serious suffering.^39^ Second, if part-human chimeras are used to generate organs or tissues for transplant, they may need to be raised in conditions inimical to animal welfare—for example, by being kept isolated from other animals of their own species.^40^ Animal welfare considerations should certainly be taken into account before research involving live-born chimeras is approved. However, it would be difficult to reject the creation of part-human chimeras on animal welfare grounds without also rejecting a wide range of existing practices that harm animals to promote human ends, including widely accepted forms of animal research and the farming of animals for meat. It could certainly be argued that we *should* radically rethink our treatment of nonhuman animals and reject the full range of these practices, but the implications of such an argument would extend far beyond part-human chimera research.

The most serious ethical concern raised by part-human chimera research is that live-born chimeras may have greater moral status than we realize, and that we may therefore treat these chimeric animals in ways that are unethical given their moral status. There is a chance that human cells could contribute to the development of the chimeric animals’ brain. A chimera with a partly humanized brain could theoretically develop a greater range of morally relevant cognitive abilities—and therefore attain higher moral status—than its nonchimeric counterparts. If a chimeric animal developed the full set of cognitive capacities we consider sufficient for full moral status, it might even have the same moral standing as a normal human adult. To kill a chimeric animal that possesses full moral status, or to experiment on it without its consent, would be morally tantamount to doing the same thing to a human.^41^

It is not implausible to think that at least some kinds of part-human chimeras may acquire morally relevant cognitive abilities. Research wherein mice were implanted with human glial cells found that the chimeric mice exhibited enhanced learning, memory, and fear conditioning.^42^ While the chimeric pig fetuses produced via blastocyst complementation by the Salk team contained only a very small proportion of human cells, it is not farfetched to think that this technique, once refined, could significantly alter the development of chimeric animals’ brains. Indeed, some areas of chimera research—such as research into human neurodegenerative disorders—would rely on the creation of chimeric animals with human-like brains.^43^ The uncertain moral status of such creatures is perhaps the most serious ethical concern raised by part-human chimera research.

Admittedly, the welfare of part-human chimeric animals would be afforded some protection under existing laws and regulations governing animal research. However, these regulations cannot fully address concerns related to part-human chimeric animals’ moral status. Moral status is widely (and we think plausibly) thought to come in degrees. The higher one's degree of moral status, the more heavily one's interests should feature in moral deliberations.^44^ Accordingly, experimenting on animals with high moral status requires stronger justification than the use of animals with lower moral status. Existing approaches to animal welfare generally do not generally take degrees of moral status into account.^45^ Moreover, regulations governing animal research tend to attach little weight to animal welfare relative to achieving valid research objectives, and therefore (at least implicitly) grant nonhuman animals a low degree of moral status.^46^ This insensitivity to degrees of moral status is arguably problematic even when such research involves non-chimeric animals, as not all animals necessarily share the same degree of moral status.^47^ The problem is especially acute in relation to chimeras with humanized brains, as such animals could theoretically possess a degree of moral status that far exceeds what is usual for their species and could, in extreme cases, even approach that of human persons.

We therefore need a regulatory framework that is sensitive not only to the welfare of part-human chimeras, but also to the prospect that at least some kinds of chimeric animals would have greater moral status than their non-chimeric counterparts. In the following section, we describe some possible legislative approaches to achieving this goal.

## POSSIBLE LEGISLATIVE APPROACHES

It would be wrong to treat a part-human chimera as if it has lower moral status than it actually possesses. Moreover, this form of wrongdoing is a legitimate target of legal intervention. Laws designed to prevent this form of wrongdoing would have the concrete aim of preventing harms to morally considerable agents; they would not merely aim to prevent victimless immoralities or ‘freefloating evils’. Even those who reject legal moralism should agree that legal coercion is permissible to prevent the infliction of morally unjustifiable harms.^48^ It is reasonable for the law to try to ensure beings are treated in accordance with their moral status, at least provided such laws are not based on unreasonable or dubious views of what confers moral status.^49^

Given the moral stakes, the law ought to specifically address the possibility of making part-human chimeras by introducing human stem cells into animal embryos (and not merely the possibility of introducing animal stem cells to human embryos). The question, then, is what form this legislation should take. At least five broad kinds of approaches are available, which we list below in order from least to most restrictive (Table 2).

**Table 2.** Possible regulatory frameworks.

Highly restrictive	• Prohibit the creation of part-human chimeric embryos, including via the introduction of human cells to animal embryos
	• Prohibit the development of part-human chimeric embryos beyond a specific threshold, such as 14 days of development or the emergence of the primitive streak
Moderately restrictive	• Allow the full development of part-human chimeras only if the human cells do not contribute to the chimeric animal's brain
	• Allow the full development of chimeras with humanized brains, but prohibit experimentation until after the chimeric animal's moral status has been determined
Highly permissive	• Do not restrict the creation of part-human chimeric embryos or live-born chimeras

The first option is to prohibit the creation of *any* part-human chimeric embryos, regardless of whether animal cells are introduced into human embryos or vice versa. This approach would straightforwardly address any ethical concerns associated with conducting such research. However, it would also rule out any possible benefits. A second option is to allow the creation of chimeric embryos but limit how far they can develop, perhaps by extending current restrictions to human embryo research to part-human embryo research. For example, we might prohibit the development of part-human chimeric embryos for longer than 14 days, beyond the emergence of the embryo's primitive streak, or beyond the onset of consciousness. The moral status of such an embryo would be no higher than that of a human embryo at an equivalent stage of development. Accordingly, those who accept research with human embryos up to a particular threshold (for example, until 14 days’ development) should presumably also accept research with part-human chimeric embryos up to an equivalent threshold. Both of these regulatory options are extremely restrictive (although the second is less restrictive than the first), and both would prevent the realization of many of the potential benefits of part-human chimera research, such as the generation of human organs for transplant. Policies that would prevent these kinds of gains require careful justification, as there are major ethical problems when a policy to ban potentially beneficial research is made on illegitimate grounds. As Savulescu argues:

There is a moral imperative to perform good research and not unnecessarily impede it. To delay by 1 year the development of a treatment that cures a lethal disease that kills 100 000 people per year is to be responsible for the deaths of those 100 000 people, even if you never see them.^50^

A third option is to allow chimeric embryos to be used to create live-born part-human chimeras *so long as human cells do not make a significant contribution to the chimeric animal's brain.* This could in principle be achieved by using gene editing to prevent human neural development in the chimeric animal.^51^ A more permissive version of this regulatory approach might only block the introduction of human pluripotent stem cells to the embryos of animals whose brain size and/or structure are already similar to those of humans, on the assumption that morally relevant characteristics would only emerge in species with brains that are already relatively humanlike.^52^ Preventing the creation of chimeras with humanized brains would straightforwardly prevent unethical experimentation on chimeras with uncertain degrees of cognitive functioning (and therefore uncertain moral status). However, blocking the creation of chimeras with substantially humanized brains would rule out some possible applications of part-human chimera research, including the use of part-human chimeras to study and develop new treatments for neurodegenerative disorders.

A fourth option is to allow the full development of chimeras with humanized brains but prohibit experimentation until after the chimera's moral status has been determined. This approach arguably strikes an ideal balance between facilitating chimera research and addressing relevant ethical concerns. In practice, however, this approach would be difficult to implement. The first difficulty is philosophical. We cannot assess chimeric animals’ moral status until we develop some justified account of what characteristics confer what degree of moral status. Unfortunately, the grounds of moral status remain a major area of debate on moral philosophy. Philosophers have defended many varying accounts of moral status, including accounts grounded in the value of sentience, rationality, autonomy, self-consciousness, empathy, and the ability to act in accordance with normative reasons. In order to determine a chimeric animal's moral status, we would first need to decide which account of moral status ought to be applied.

The second difficulty is epistemological. Once we have developed a plausible account of moral status, we will need to screen part-human chimeras for morally relevant capacities. These capacities will be difficult to detect when they occur in part-human chimeras as we cannot converse with non-human animals directly, while inferences drawn from animal behavior or physiology are open to question. Exactly what form this testing should take would depend on what characteristics we believe are morally relevant. Plausibly, however, behavioral investigations should investigate the diverse range of ways that chimeric animals might display morally relevant characteristics, including by evaluating various forms of functioning across a range of social arrangements; if a chimeric animal fails to display species-atypical abilities on *one* test, this should not be taken as conclusive evidence that it lacks morally relevant characteristics altogether. Because it would be gravely unethical to harm a chimeric animal with full moral status, we should generally err on the side of overestimating moral status rather than underestimating it. Given the moral stakes, part-human chimeras should arguably be treated in accordance with the highest level of moral status they might realistically possess.^53^

The final option is to leave the creation of part-human chimeras by introducing human cells to animal embryos outside the scope of existing legislation. It is sometimes argued that existing loopholes *should* be left open in order to allow valuable forms of chimera research to occur in jurisdictions with otherwise strict laws on human/animal hybrids and chimeras.^54^ However, leaving such loopholes open may fail to prevent practices that are highly unethical, such as harmful experimentation on live-born chimeras with human-like cognitive capacities. We should leave these legislative loopholes open only if alternative forms of oversight could serve equally well—a possibility we consider in the following section of this paper.

On balance, we believe the third and fourth approaches are the most promising. Both would allow the creation of live-born human-nonhuman primary chimeras (with the attendant scientific benefits) while protecting against the most pressing ethical concerns. The third option would more fully address concerns about ‘morally humanized’ chimeras, but it would also rule out *all* research involving chimeras with substantially humanized brains—including in cases where these chimeras have not acquired any cognitive capacities that could plausibly increase their moral status. The fourth option is perhaps ideal, as it would block only those forms of research involving unethical experimentation with live-born chimeras. However, in order to implement this approach we would first need to develop a justified account of moral status and work out how to apply it. Such an approach would therefore require us to resolve difficult philosophical questions about what characteristics confer moral status and how these characteristics can be identified in part-human chimeric animals.^55^ It would also require us to work out how to deal with cases of uncertainty regarding moral status; as discussed above, we think is best done by erring on the side of overestimating rather than underestimating moral status.

## ALTERNATIVE FORMS OF OVERSIGHT

So far, we have focused on legislative solutions to concerns about chimeric animals’ moral status. This is because the creation of part-human chimeric embryos is—in Australia and elsewhere—already a matter of statutory law. The fact that such laws apply only if the embryo is created by adding *animal* cells to *human* embryos is a peculiarity that seems, in part, to reflect a lack of scientific foresight at the time such laws were drafted. The most obvious way to address this peculiarity is to revise these laws so they can also address the unique ethical issues associated with creating part-human chimeras by introducing human cells to animal embryos.

However, this is not the only possible approach. Other regulatory mechanisms could potentially achieve the same end (see Table 3). For example, government agencies that fund scientific research could prohibit funding for certain categories of chimera research. In some jurisdictions, this approach has effectively prevented any chimera research from taking place unless it accords with funding agencies’ guidelines, even if such research would be permitted under the relevant legislation.^56^ While such restrictions can be effective, their power is not absolute. Notably, the recent creation of human-pig chimeric fetuses at the Salk institute was funded largely by private foundations, and so was able to proceed despite the National Institute of Health's moratorium on funding part-human chimera research.^57^

**Table 3.** Alternatives to legislative reform.

Regulatory mechanism	Potential role
Restrictions on public funding	Agencies responsible for funding stem cell research could prohibit categories of research that are not prohibited by legislation, effectively preventing such research unless (and only insofar as) this research can be funded privately.
Ethics committee oversight	Ethics committee oversight can prevent unethical practices that are not prohibited by legislation. However, ethics committees’ ability to perform this function might depend, in part, on whether relevant ethical guidance is available for such committees to draw on.
Professional guidelines	Professional guidelines can inform regulation on multiple levels, including by influencing the development and interpretation of legislation, the activities of funding agencies, and the deliberations of research oversight committees.

Another option is to rely on oversight committees to approve or reject specific research projects involving the creation of part-human chimeras with potentially humanized brains.^58^ This oversight process could be performed at a number of different levels, ranging from national review to local institutional review.^59^ On any such model, it is important that scientists, bioethicists, and others develop a moral framework for part-human chimera research ahead of time; otherwise, there is a risk that oversight committees will be forced to make reactive, knee-jerk judgments when faced with applications for specific projects.

The International Society for Stem Cell Research (ISSCR) has already proposed a set of ethical standards for part-human chimera studies to help guide the deliberations of ethics committees. This document explains how existing principles of animal welfare can be applied to the unique challenges raised by part-human chimera research. Among other suggestions, the ISSCR guidelines highlight the possibility that chimeric animals with humanized brains might have different welfare needs to their non-chimeric counterparts, suggest that it may be appropriate to screen for deviations from species-typical norms that could be relevant to chimeric animals’ welfare, and hold that research with a significant potential to create humanized cognition or awareness should be subject to especially close scrutiny to ensure the ethical protection of animal research subjects.^60^ These principles focus on respecting animal welfare in this novel area of scientific inquiry.^61^

While useful, these recommendations leave some crucial questions open. The ISSCR standards are intended to help ethics committees apply existing principles of animal welfare to part-human chimeras. They do not explicitly consider how such research could affect the moral status of chimeric animals (which could render unethical certain forms of experimentation that it would have been ethical to perform using animals of lower moral status)—concerns that may be still speculative, but which we don’t think should be dismissed out of hand. Even if, in principle, ethics committee oversight could address these concerns (and thereby render legislative reform unnecessary), they cannot effectively fulfill this function until the outstanding philosophical questions regarding chimeric animals’ moral status have been resolved and some broad moral limits to part-human chimera research have been identified.

## CONCLUSION

The creation of part-human chimeric embryos and live-born chimeras could prove enormously beneficial as a tool for studying development and disease, testing therapeutic drugs, and generating tissues and organs for transplant. Although many jurisdictions prohibit the creation of some kinds of chimeric embryos outright, the creation of chimeric embryos by introducing human stem cells to animal embryos often falls within the gaps of the relevant legislation. We should rethink these laws, or, failing that, we should introduce alternative forms of regulation that can prevent unethical forms of chimera research. We should not ignore important ethical concerns about chimeric animals’ moral status, but nor should we unnecessarily foreclose the potential benefits of this area of research. Instead, we will need to work out how best to balance two key goals: to facilitate ethical research involving part-human chimeras and to prevent unethical experimentation with chimeras that have an uncertain—and potentially substantial—degree of moral status.

## Footnotes

^1^ Chimeras, so defined, are distinct from other kinds of ‘inter-species’ entities such as hybrids and genetically altered animals. Hybrids are formed from an egg from one species and sperm from another, whereas genetically altered animals (including transgenic animals) are created by inserting one or more genes from one species into another. Hybrids and genetically altered animals would have a mixture of human and animal DNA. Chimeras, by contrast, are composed of a mix of human and animal cells. Academy of Medical Sciences, *Animals**Containing**Human**Material* §2.2 (2011), https://acmedsci.ac.uk/policy/policy-projects/animals-containing-human-material (last accessed Aug. 10, 2018).

^2^ Tamir Rashid, Toshihiro Kobayashi & Hiromitsu Nakauchi, *Revisiting the**Flight**of Icarus:**Making**Human**Organs**from PSCs with**Large**Animal**Chimeras*, 15 Cell Stem Cell 406 (2014).

^3^ Toshihiro Kobayashi et al., *Generation of**Rat**Pancreas**in**Mouse**by**Interspecific**Blastocyst**Injection**of**Pluripotent**Stem**Cells*, 142 Cell 787 (2010).

^4^ Tomoyuki Yamaguchi et al., *Interspecies**Organogenesis**Generates**Autologous**Functional**Islets*, 542 Nature 191 (2017).

^5^ Jun Wu et al., *Interspecies Chimerism with Mammalian Pluripotent Stem Cells*, 168 Cell 473 (2017).

^6^ Jun Wu et al., *Generation of**Human**Organs**in**Pigs**via**Interspecies**Blastocyst**Complementation*, 51 Reprod. Domest. Anim. 18 (2016).

^7^ Nuffield Council on Bioethics, *Animal-to-**Human**Transplants**:**The**Ethics**of**Xenotransplantation* (1996), http://nuffieldbioethics.org/wp-content/uploads/xenotransplantation.pdf (last accessed Nov. 15, 2018)*.*

^8^ The ethical issues raised by interspecies blastocyst complementation could also intersect with long-running debates about human embryo research if the cells used are derived from human embryos. However, insofar as non-embryonic (e.g. induced pluripotent) stem cells can be used instead, these issues are not essential to part-human chimera research.

^9^ Prohibition of Human Cloning for Reproduction Act 2002 (Cth).

^10^
*Id.* §8.1.

^11^
*Id.*

^12^ Nita A. Farahany et al., *The**Ethics**of**Experimenting**with**Human**Brain**Tissue*, 556 Nature 429 (2018).

^13^
*Id.* at 342.

^14^ Abena Hagan-Brown et al., *Newspaper**Coverage**of**Human**-**Pig**Chimera**Research**: A**Qualitative**Study**on**Select**Media**Coverage**of**Scientific**Breakthrough*, 24 Xenotransplantation e12317 (2017).

^15^ Rodolphe Bourret et al., *Human–**Animal**Chimeras**:**Ethical**Issues**about**Farming**Chimeric**Animals**Bearing**Human**Organs*, 7 Stem Cell Res Ther 87 (2016); Henry T. Greely, *Human/**Nonhuman**Chimeras**: Assessing the**Issues*, *in* 671 The Oxford Handbook of Animal Ethics (Tom L. Beauchamp & R. G. Frey eds., 2013); Phillip Karpowicz et al., *Developing**Human**-**Nonhuman**Chimeras**in**Human**Stem**Cell**Research**: Ethical**Issues**and**Boundaries*, Kennedy Inst. Ethics J 535 (2005); David Shaw et al., *Creating**Human**Organs**in**Chimaera**Pigs**:**An**Ethical**Source**of**Immunocompatible**Organs**?*, 41 J. Med. Ethics 970 (2015); Hiroshi Mizuno et al., *Ethical**Acceptability**of**Research**on**Human**-**Animal**Chimeric**Embryos**:**Summary**of**Opinions**by the Japanese Expert Panel on Bioethics*, 11 Life Sci. Soc. Pol’y 15 (2015); Robert Streiffer, *At the**Edge**of**Humanity**:**Human**Stem**Cells**,**Chimeras**, and**Moral**Status*, 15 Kennedy Inst. Ethics J. 347 (2005).

^16^ Prohibition of Human Cloning for Reproduction Act 2002, *supra* note 9.

^17^ Assisted Human Reproduction Act, S.C. 2004, C.2

^18^ Franc¸oise Baylis & Jason Scott Robert, *Part-Human Chimeras: Worrying the Facts, Probing the Ethics*, 7 Am. J. Bioeth. 41, 42 (2007); Natural Sciences and Engineering Research Council of Canada Canadian Institutes of Health Research, and Social Sciences and Humanities Research Council of Canada, *Tri-Council Policy Statement: Ethical Conduct for Research Involving Humans* (2014), http://www.pre.ethics.gc.ca/pdf/eng/tcps2-2014/TCPS_2_FINAL_Web.pdf (last accessed Aug. 10, 2018).

^19^ Gretchen Vogel, *Major**Grant**in**Limbo**. NIH**Revisits**Ethics**of**Animal**-**Human**Chimeras*, 350 Science 261 (2015).

^20^ National Research Council (US) and Institute of Medicine (US) Human Embryonic Stem Cell Research Advisory Committee, *National Academies' Guidelines for Human Embryonic Stem Cell Research Amended as of May 2010, §7.3* (2010), https://www.nap.edu/read/12923/ (last accessed Aug. 10, 2018).

^21^ Human Chimera Prohibition Act, S.1373, 109th Cong. (2005).

^22^ Human-Animal Chimera Prohibition Act, H.R. 6131, 114th Cong. (2016).

^23^ Human Fertilisation and Embryology Act, c. 220, §4A (2008).

^24^ Academy of Medical Sciences, *supra* note 1, at 9.

^25^ Mizuno et al., *supra* note 15, at 3.

^26^
*Id.*, at 5.

^27^ H. M. Beier & J. O. Beckman, *German Embryo Protection Act (October 24^th^, 1990): Gesetz zum Schutz von Embryonen (Embryonenschutzgesetz-ESchG)*, 6 Hum. Reprod. 605 (1991)

^28^ German Ethics Council, *Human–Animal Mixtures in Research* 111–17 (2011), https://www.ethikrat.org/fileadmin/Publikationen/Stellungnahmen/englisch/opinion-human-animal-mixtures-in-research.pdf (last accessed Aug. 10, 2018).

^29^ Bourret et al., *supra* note 15, at Table 1; Chloe Giquel, et al., *The**Creation**of**Chimeric**Animals**Carrying**Human**Organs*, 137 Med. & Law 37–47 (2016).

^30^ David Shaw, *Creating Chimeras for Organs Is Legal in Switzerland*, 7 BioethicaForum 24 (2014).

^31^ International Society for Stem Cell Research, *Guidelines for Stem Cell Research and Clinical Translation* §2.1.3 (2016), http://www.isscr.org/docs/default-source/all-isscr-guidelines/guidelines-2016/isscr-guidelines-for-stem-cell-research-and-clinical-translation.pdf (last accessed Nov. 25, 2018).

^32^
*Id.,* at §2.1.5.

^33^ Ruth F. Chadwick, *Playing God*, 3 Cogito 186–93 (1989).

^34^ Jason Scott Robert & Françoise Baylis, *Crossing**Species**Boundaries*, 3 Am. J. Bioeth. 1–13 (2003).

^35^ Julian Savulescu, *Genetically**Modified**Animals**: Should**There**Be**Limits**to**Engineering**the**Animal**Kingdom**?*, *in* 641 The Oxford Handbook of Animal Ethics 651–52 (Tom L. Beauchamp & R. G Frey eds., 2013); Michael Tooley, *Personhood, in* 127 A Companion to Bioethics (Helga Kuhse & Peter Singer eds., 2nd ed., 2010)

^36^ For example, if livestock animals have a non-trivial degree of moral status, the harm inflicted on such animals by current methods of meat production might constitute a serious moral evil that urgently needs to be addressed. See Alastair Norcross, *Puppies, Pigs, and People: Eating Meat and Marginal Cases,* 18 Phil. Persp. 229–45(2004);Peter Singer, Animal Liberation (2009); Robert Streiffer, *Human/Non-Human Chimeras, in*Stanford Encylopedia of Philosophy §3 (Edward N. Zalta, ed. 2015).

^37^ César Palacios-González, *Ethical**Aspects**of**Creating**Human**-**Nonhuman**Chimeras**Capable**of**Human**Gamete**Production**and**Human**Pregnancy**,* 33 MonashBioeth. Rev. 181 (2015).

^38^ Greely, *supra* note 15, at 686.

^39^ Insoo Hyun, *What's Wrong with Human/Nonhuman Chimera Research?*, 14 PLoSbiol. e1002535 (2016).

^40^ Shaw et al., *supra* note 15, at 973.

^41^ Streiffer, *supra* note 15, at 362.

^42^ Xiaoning Han et al., *Forebrain**Engraftment**by**Human**Glial**Progenitor**Cells**Enhances**Synaptic**Plasticity**and**Learning**in**Adult**Mice*, 12 Cell Stem Cell 342 (2013).

^43^ Françoise Baylis & Andrew Fenton, *Chimera**Research**and**Stem**Cell**Therapies**for**Human**Neurodegenerative**Disorders*, 16 Camb. Q. Healthc. Ethics 195 (2007); Sonya Levine & Laura Grabel, *The**Contribution**of**Human**/**Non**-human**Animal**Chimeras**to**Stem**Cell**Research**,* 24 Stem Cell Res 128 (2017).

^44^ David DeGrazia, *Moral Status as a Matter of Degree?,* 46 S. J. Phil. 181 (2008)

^45^ Special restrictions on primate research may be one exception. Marie Fox, *The**Legal**Regulation**of**Primate**Research**,* 9 Am. J. Bioethics 13 (2009).

^46^
Gary Francione, Animals, Property & The Law (1995); Streiffer, *supra* note 15, at 362.

^47^ Shally Kagan, *For Hiera**r**chy in Animal Ethics,* 6 J. Pract. Ethics 1 (2018);Jan Lauwryens, Rethinking the Three R’s in Animal Research §3.2.2 (2018).

^48^
Joel Feinberg, The Moral Limits of the Criminal Law Volume 1: Harm to Others (1987).

^49^ Robert Streiffer, *Chimeras, Moral Status, and Public Policy: Implications of the Abortion Debate for Public Policy on Human/Nonhuman Chimera Research*, 38 J. L. Med. & Ethics 238 (2010).

^50^ Julian Savulescu, *Bioethics: Why Philosophy is Essential for Progress.* 41 J. Med. Ethics 28 (2015), at 31; see also Streiffer, *supra* note 49.

^51^ Rashid et al., *supra* note 2.

^52^ Greely, *supra* note 15, at 682–83; Karpowicz et al., *supra* note 15, at 124–26, 128–29.

^53^ Savulescu, *supra* note 35, at 653.

^54^ Shaw, *supra* note 30, at 26.

^55^ See e.g. Streiffer, *supra* note 15, at 363–66.

^56^ Baylis & Robert 2007, *supra* note 18, at 42; Andrea Bonnicksen, Chimeras, Hybrids, and Interspecies Research 50–51 (2009).

^57^ Sharon Begley, *First**Human**-**Pig**Chimeras**Created**,**Sparking**Hopes**for**Transplantable**Organs**– and**Debate**,*STAT (2017), https://www.statnews.com/2017/01/26/first-chimera-human-pig/ (last accessed Nov. 26, 2018).

^58^ National Research Council (US) and Institute of Medicine (US) Human Embryonic Stem Cell Research Advisory Committee, *supra* note 20, at §7.3

^59^ Some argue that national review offers distinct advantages over local review, including that such a model would limit bias, provide more consistent outcomes, and facilitate expert review in contexts where researchers with appropriate expertise are not be available at the local level. See Francoise Baylis & Jason Scott Robert, *Human**Embryonic**Stem**Cell**Research**:**An**Argument**for**National**Research**Review**,* 13 Account. Res. 207 (2006); Baylis & Robert, *supra* note 18, at 43.

^60^ Insoo Hyun et al., *Ethical Standards for Human-To-Animal Chimera Experiments in Stem Cell Research*, 1 Cell Stem Cell 159 (2007).

^61^
*Id*. See also Hyun, *What's Wrong with Human/Nonhuman Chimera Research?* 14 PLoSBiol. e1002535 (2016).

